# Analysis of a Remote Monitoring Program for Symptoms Among Adults With Cancer Receiving Antineoplastic Therapy

**DOI:** 10.1001/jamanetworkopen.2022.1078

**Published:** 2022-03-04

**Authors:** Bobby Daly, Kevin Nicholas, Jessica Flynn, Nicholas Silva, Katherine Panageas, Jun J. Mao, Lior Gazit, Dmitriy Gorenshteyn, Stefania Sokolowski, Tiffanny Newman, Claire Perry, Isaac Wagner, Alice Zervoudakis, Rori Salvaggio, Jessie Holland, Yeneat O. Chiu, Gilad J. Kuperman, Brett A. Simon, Diane L. Reidy-Lagunes, Wendy Perchick

**Affiliations:** 1 Memorial Sloan Kettering Cancer Center, New York, New York

## Abstract

**Importance:**

Electronic patient-reported outcomes (ePROs) may have the potential to improve cancer care delivery by enhancing patient quality of life, reducing acute care visits, and extending overall survival. However, the optimal cadence of ePRO assessments is unknown.

**Objective:**

To determine patient response preferences and the clinical value associated with a daily cadence for ePROs for patients receiving antineoplastic treatment.

**Design, Setting, and Participants:**

This quality improvement study of adult patients undergoing antineoplastic treatment assessed a remote monitoring program using ePROs that was developed to manage cancer therapy-related symptoms. ePRO data submitted between October 16, 2018 to February 29, 2020, from a single regional site within the Memorial Sloan Kettering Cancer Center network were included. Data were analyzed from April 2020 to January 2022.

**Exposure:**

While undergoing active treatment, patients received a daily ePRO assessment that, based on patient responses, generated yellow (moderate) or red (severe) symptom alerts that were sent to clinicians.

**Main Outcomes and Measures:**

The main outcomes assessed included patient response rate, symptom alert frequency, and an analysis of the clinical value of daily ePROs.

**Results:**

A total of 217 patients (median [range] age, 66 [31-92] years; 103 [47.5%] women and 114 [52.5%] men) initiating antineoplastic therapy at high risk for symptoms were monitored for a median (range) of 91 (2-369) days. Most patients had thoracic (59 patients [27.2%]), head and neck (48 patients [22.1%]), or gastrointestinal (43 patients [19.8%]) malignant neoplasms. Of 14 603 unique symptom assessments completed, 7349 (50.3%) generated red or yellow symptom alerts. Symptoms commonly generating alerts included pain (665 assessments [23.0%]) and functional status (465 assessments [16.1%]). Most assessments (8438 assessments [57.8%]) were completed at home during regular clinic hours (ie, 9 am-5 pm), with higher response rates on weekdays (58.4%; 95% CI, 57.5%-59.5%) than on weekend days (51.3%; 95% CI, 49.5%-53.1%). Importantly, 284 of 630 unique red alerts (45.1%) surfaced without a prior yellow alert for the same symptom within the prior 7 days; symptom severity fluctuated over the course of a week, and symptom assessments generating a red alert were followed by an acute care visit within 7 days 8.7% of the time compared with 2.9% for assessments without a red alert.

**Conclusions and Relevance:**

These findings suggest that daily ePRO assessments were associated with increased insight into symptom management in patients undergoing antineoplastic treatment and symptom alerts were associated with risk of acute care.

## Key Points

**Question:**

What is the clinical value associated with a daily cadence of electronic patient-reported outcomes (ePROs) symptom assessments for patients with cancer initiating antineoplastic treatment?

**Findings:**

This quality improvement study including 217 patients found that severe symptoms frequently emerged without prior signs and that symptoms fluctuated over the course of a week. Severe symptom alerts were also associated with acute care events.

**Meaning:**

These findings suggest that there is potential value of a daily cadence for ePRO assessments; a less frequent cadence may miss rapidly developing symptoms that require prompt management.

## Teaser

This quality improvement study assesses the clinical value of a daily cadence of electronic patient-reported outcomes for patients undergoing antineoplastic treatment.

## Introduction

A patient-reported outcome describes “the impact of health-related conditions and/or treatments provided by patient self-report without introduction of bias by any third party.”^1,2^ Electronic patient reported outcomes (ePROs) can improve cancer care delivery by enhancing quality of life, reducing acute care visits, and improving overall survival.^3,4,5,6,7^ Patient groups, clinicians, and policy makers have advocated to make ePROs the standard of care for patients receiving active cancer treatment. Furthermore, the Center for Medicare & Medicaid Services Center for Medicare and Medicaid Innovation proposed ePROs as a required feature for the successor to the Oncology Care Model.^8^ Incorporation of ePROs in clinical practice is widely viewed as fundamental to improving care delivery for patients with cancer.

Despite broad support, sizeable implementation issues remain related to ePROs use. In oncologic care, ePROs are generally collected using brief electronic surveys with alerts to clinicians for concerning symptoms.^9^ Enhanced patient-clinician communication is thought to underlie outcome improvements associated with ePRO assessments. To realize this benefit, many institutions are integrating ePROs into clinical workflows, and private companies have emerged to assist with the technology and integration efforts. Yet, as oncology practices build these systems, there is a risk for failure if key components of successful ePRO implementation are not identified and incorporated.

Several essential ePRO system components have been established: (1) a focus on relevant symptoms, (2) systematic repeated assessments, and (3) clinician engagement.^10^ An open question is the “optimal dose” of assessments. Selecting dose or ePRO cadence is crucial because ePRO assessments can be a burden for patients to complete and clinicians to interpret and can impact the workflow and workforce necessary for proper monitoring.^9,11,12^ ePRO cadences have varied from daily,^6^ to weekly,^4,5,13^ to a prespecified period postantineoplastic treatment,^14,15^ to coordinated with clinic visits,^16^ with no consensus around optimal timing. Patients and clinical stakeholders are interested in determining the optimal ePRO cadence in cancer care. Recent studies evaluating large-scale ePRO implementation in community oncology practices have deployed with weekly cadences based on the National Cancer Institute PRO version of the Common Terminology Criteria for Adverse Events (PRO-CTCAE).^11,12,17^ The PRO-CTCAE uses a recall period of the last 7 days (eg, “In the last 7 days, how OFTEN did you have PAIN?”) based on the practical considerations that daily assessments would not be feasible in most cancer clinical trials and a longer recall period would lead to memory degradation and loss of symptom information.^17^ In contrast, we launched a clinical program, InSight Care, that provided daily ePROs to patients initiating antineoplastic therapy who were at a high risk for toxic effects to allow real-time symptom monitoring.^7,18^ We chose daily ePRO assessments to test the hypotheses that symptom assessments would be submitted more frequently during clinic office hours (ie, 9 am-5 pm) than at off-hours, would be reduced during weekends compared with weekdays, and that response rates would not differ based on duration of monitoring. We also analyzed the symptom alert data to determine whether daily monitoring of patients’ symptoms provided valuable clinical information, including variation in symptoms and association with acute care.

## Methods

This quality improvement study received a waiver of review and informed consent from the Memorial Sloan Kettering Cancer Center (MSK) institutional review board because it was a quality improvement study. This study is reported following the Revised Standards for Quality Improvement Reporting Excellence (SQUIRE 2.0) reporting guideline.^19^

### Program Description and Participants

Our ePRO program was launched on October 15, 2018, at an MSK ambulatory care site. This technology-enabled program identifies patients initiating antineoplastic therapy who are at high risk for treatment-related symptoms, monitors the symptoms of enrolled patients daily via an ePRO assessment, and intervenes as necessary to mitigate reported symptoms. Adult patients with solid tumors or lymphoma initiating intravenous antineoplastic therapy for the first time who had access to a smartphone, tablet, or computer and who were identified by their oncologist as high-risk for acute care were eligible. Race was determined by patient self-report and was captured to better understand the generalizability of the results. A machine learning model described elsewhere^20^ supported high-risk classifications. A dedicated team of oncology registered nurses and nurse practitioners monitored and managed reported symptoms through a digital platform (eFigure 1 in the Supplement).^7^ Patients exited the program when they were no longer on active treatment. Program details are presented elsewhere.^7,21,22^

### Symptom Assessment and Symptom Alerts

At treatment initiation, patients completed program onboarding consisting of a program overview, an introduction to the technology interface, and education on the role of ePROs in their care. Enrolled patients subsequently received daily symptom assessments accessed through the MSK Patient Portal (a Health Insurance Portability and Accountability Act of 1996–adherent, web-accessible communication and patient data system) and could complete the assessment using devices with internet or cellular access. There was no automated trigger or phone call to remind patients to complete the assessments. Survey questions were adapted from the PRO-CTCAE and the ongoing Electronic Patient Reporting of Symptoms During Cancer Treatment (PRO-TECT) trial.^23,24^ Assessments asked about common symptoms for patients on antineoplastic treatment that drive acute care visits, including pain, functional status, anorexia or dehydration, nausea, vomiting, diarrhea, and dyspnea (eAppendix in the Supplement).^25,26^ The survey was designed for a 1-time daily interaction ranging from less than 1 minute to several minutes, depending on the patient’s symptom burden.^7^ Logic was developed to generate alerts, including red for severe symptoms and yellow for mild or moderate symptoms. For example, for the question “How often did you have nausea in the last 24 hours?” a red alert response would be almost all the time or all the time and a yellow alert response would be some of the time. Alerts could be generated for each symptom, so a single assessment could yield multiple yellow and red symptom alerts.

### Alert Prevalence

ePRO data were evaluated from October 16, 2018, to February 29, 2020; this cutoff was chosen owing to program changes after this date caused by the COVID-19 pandemic.^27,28^ The total number of assessments generating alerts and alert severity (red or yellow) by symptom were quantified.

### Patient Revealed Preference for ePROs

Symptom assessments could be completed once per 24-hour period and were available 24 hours a day, 7 days a week. Response rate was defined as the percentage of enrolled days patients completed the symptom assessment. Preference for completing assessments on weekdays vs weekends was assessed, along with intraday response profiles. To evaluate weekday vs weekend preference, we compared response rates between weekdays (Monday-Friday) and weekend (Saturday and Sunday) days. To evaluate time of day preference, we looked at the distribution of survey completion over 24 hours to assess preferred times of day. To understand how response rates varied with time in the program, we compared mean response rates based on elapsed enrollment week.

### Cadence Questions

We formulated 3 a priori questions to evaluate whether a daily cadence is of clinical value for this patient population and what symptom data would be lost if patients were monitored with a weekly rather than daily ePRO assessment. We adhered to the structure recommended by SQUIRE 2.0 for quality improvement studies of identifying the problem or outstanding question, the rationale to explain the problem, the proposed methods to answer that question, and the subsequent results of that analysis.^19^ The analysis was conducted at the assessment and symptom alert level.

Our first question was what proportion of red alerts were generated without a preceding yellow alert? The rationale was that if a patient has a severe symptom without a preceding moderate symptom, a less frequent cadence could contribute to patient discomfort by missing severe symptom days. To assess this question, we used alert comparisons at the symptom level. We considered completed assessments in the 7 days prior to the red alert to evaluate what clinical information would be lost in shifting from daily to weekly assessments. Only assessments with a red alert for which at least 1 other assessment was completed in the 7 days prior were included. If a patient had multiple red alerts for the same symptom in a 7-day period, we looked back the 7 days prior to the first red alert for that symptom to avoid duplication. From this subset, we evaluated what proportion of red alerts did not have a yellow alert for the same symptom in the 7 days prior.

Our second question was how does symptom severity change over a week? Our rationale was that if symptom severity minimally fluctuates over a week, then a daily cadence might not provide value. To assess this question, analysis was at the symptom level, employed fixed 7-day windows (eg, 1-7, 8-14, 15-21), and created static time boxes for patients, evaluating symptom reporting and alerts in that period. To ensure a fair comparison of alert status changes, we required that each 7-day window contained at least 2 completed assessments. The 6 scenarios evaluated included no alert to yellow alert, no alert to red alert, yellow alert to red alert, yellow alert to no alert, red alert to yellow alert, and red alert to no alert. For each time box and symptom, we evaluated the frequency of the above scenarios. Lastly, we evaluated what proportion of time windows had any fluctuation (ie, involved any of these scenarios).

Our third question was are alerts associated with future acute events? For this question, our rationale was that a signal in the daily ePRO alerts would enable the clinical team to provide anticipatory care and could be used for future prognostic analytics. To assess this, we used forward-looking time boxes, examining assessments with a red alert to see if an acute care event occurred in the following 7 days. This analysis was at the assessment level. Acute events (unanticipated emergency department visits or inpatient admissions) were captured in our electronic health record. We evaluated how often days when patients reported red alert symptoms (considered red alert days) were associated with acute events within 7 days compared with days with no red alerts and further evaluated how this risk may change based on red alert count per assessment (eg, red alerts for multiple symptoms, such as pain and diarrhea, on a single assessment).

### Data Analysis

Analyses were performed in R statistical software version 3.6.3 (R Project for Statistical Computing). Data were analyzed from April 2020 to January 2022.

## Results

Between October 16, 2018, and February 29, 2020, a total of 217 patients (median [range] age, 66 [31-92] years; 103 [47.5%] women and 114 [52.5%] men) enrolled in the ePRO program at the start of intravenous antineoplastic treatment. Of these, 10 patients (4.6%) were Asian, 15 patients (6.9%) were Black, and 186 patients (85.7%) were White (Table 1). The most common cancer diagnoses were thoracic (59 patients [27.2%]), head and neck (48 patients [22.1%]), and gastrointestinal (43 patients [19.8%]).

**Table 1.** Sociodemographic and Clinical Characteristics of Enrolled Patients

Characteristic	Enrolled patient population (N = 217)
Age, median (range), y	66 (31-92)
Sex	
Women	103 (47.5)
Men	114 (52.5)
Race	
Asian	10 (4.6)
Black	15 (6.9)
White	186 (85.7)
Missing or unknown	6 (2.8)
Marital status	
Divorced	14 (6.5)
Partner	151 (70.6)
Separated	3 (1.4)
Single	28 (12.9)
Widowed	20 (9.2)
Unknown	1 (0.5)
Cancer	
Breast	25 (11.5)
Head and neck	48 (22.1)
Gastrointestinal	43 (19.8)
Genitourinary	23 (10.6)
Gynecology	7 (3.2)
Lymphoma	6 (2.8)
Melanoma	5 (2.3)
Thoracic	59 (27.2)
Soft tissue	1 (0.5)
Initial intravenous antineoplastic agent	
Chemotherapy only (includes cytotoxic, antibodies)	167 (76.9)
Immunotherapy only	22 (10.1)
Combination chemotherapy and immunotherapy	28 (12.9)
Radiation within 14 d of antineoplastic	28 (12.9)
Treatment intent	
Curative	102 (47.0)
Palliative	115 (53.0)

**Figure 1.**
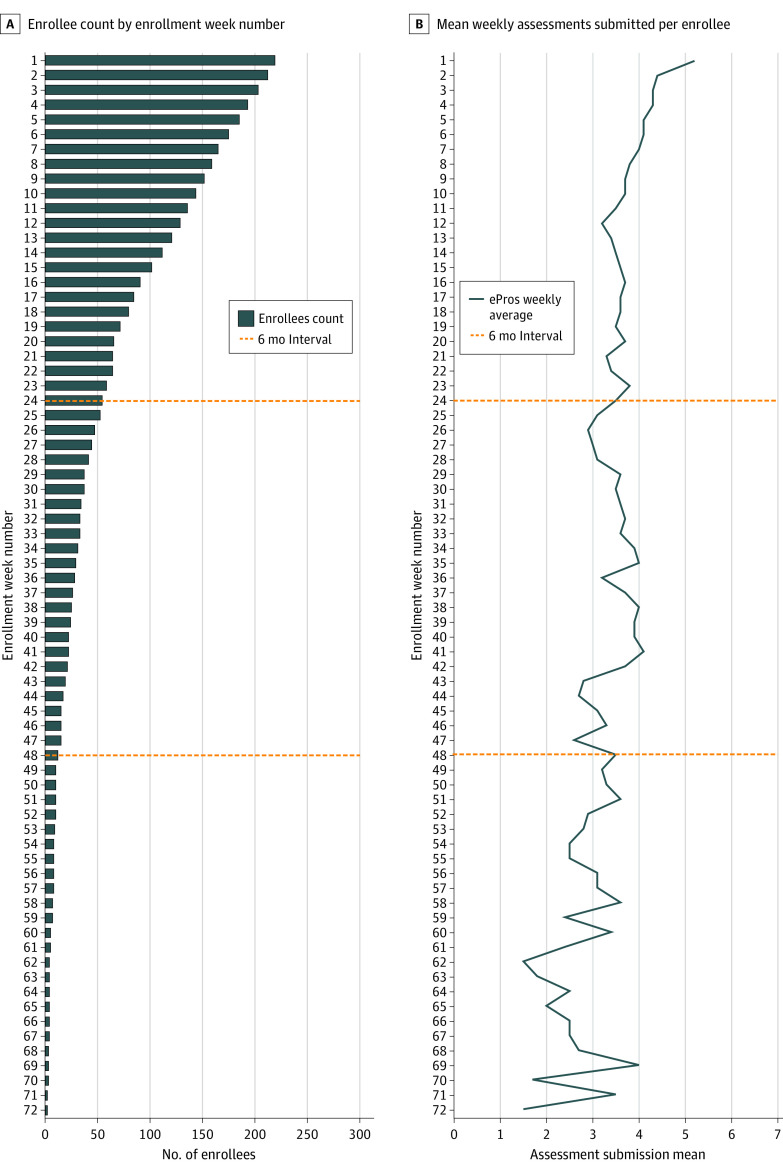
Count of Patient Enrollees by Week Number and Mean Weekly Electronic Patient-Reported Outcomes (ePROs) Assessment Submission by Enrollment Week

During the study, 14 603 unique symptom assessments were completed. The median (range) enrollment was 91 (2-369) days. Enrolled patients completed a mean (SD) of 3.9 (2.5) assessments per week from day 1 to 6 months, 3.5 (2.5) assessments per week from 6 months to 1 year, and 2.7 (2.1) assessments per week after 1 year of enrollment (Figure 1).

**Figure 2.**
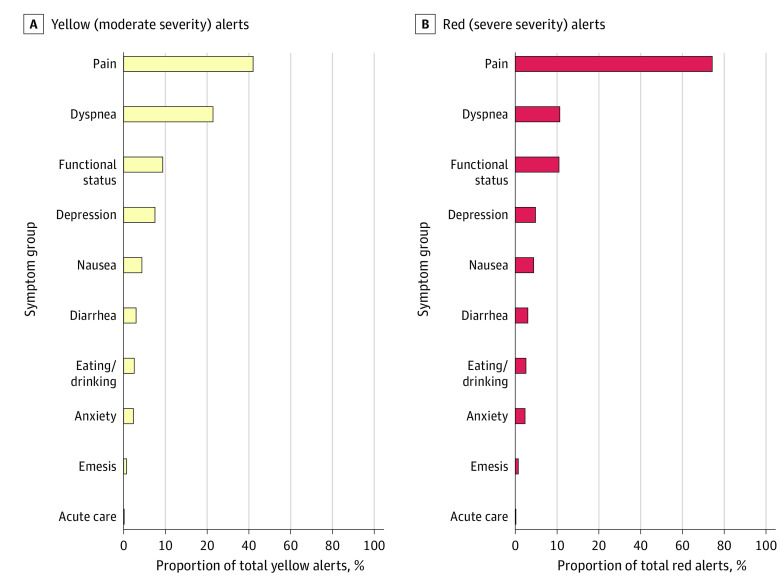
Yellow and Red Alerts by Symptom

Approximately half of all completed assessments (7254 assessments [49.7%]) generated no alerts; 6998 assessments (47.9%) generated at least 1 yellow alert, 2648 assessments (18.1%) generated at least 1 red alert, and 2297 assessments (15.7%) generated yellow and red alerts. The assessments generated a total of 23 112 symptom alerts, including 17 505 yellow alerts (75.7%) and 5607 red alerts (24.3%). Pain, dyspnea, and functional status were the most prevalent symptoms to generate yellow and red alerts (Figure 2).

**Figure 3.**
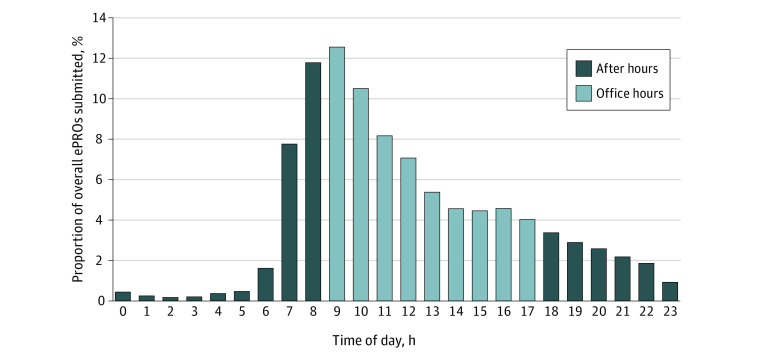
Electronic Patient-Reported (ePROs) Outcomes Assessment Completion by Hour of Day

The response rate on weekdays (58.4%; 95% CI, 57.5%-59.5%) was higher than on weekends (51.3%; 95% CI, 49.5%-53.1%). Most assessments (8438 assessments [57.8%]) were completed between 9 am and 5 pm, during regular office hours (Figure 3); 7 am to 9 am (2959 assessments [20.3%]) and 5 pm to 9 pm (2305 assessments [15.8%]) were the most common times assessments were completed outside of office hours.

### Cadence Questions

#### Red Alerts Generated Without a Preceding Yellow Alert

Of 3519 assessments that generated red alerts, 951 (27.0%) had no prior red alert for the same symptom within the 7 days prior. Of those 951 red alert assessments, 630 (66.2%) had a prior symptom assessment submitted within 7 days and were analyzed. Of these, 284 assessments (45.1%) did not have a yellow alert for the same symptom in the 7 days prior indicating that the severity of the symptom arose de novo.

#### Changes in Symptom Severity in 7-Day Windows

There were 2896 seven-day fixed time box windows containing at least 2 assessments. Of these, 1745 time boxes (60.3%) showed an alert fluctuation in at least 1 symptom. Most fluctuations were from no alert to yellow alert or vice-versa. Symptoms with the highest fluctuation rates within a time box were those with the highest number of red and yellow alerts and included pain (665 assessments [23.0%]) and functional status (465 assessments [16.1%]) (eFigure 2 and eTable in the Supplement).

#### Association of Alerts With Future Acute Events

During the evaluated enrollment period, 77 patients (35.5%) had an acute event, and there were 121 acute events. Symptom assessments generating a red alert (2648 unique red alert days) were followed by an acute care event within 7 days for 231 assessments (8.7%), this compares with symptom assessments generating no red alert (11 955 unique no red alert days) that were followed by an acute event within 7 days for 349 assessments (2.9%). Thus, if a patient submitted an assessment with a red alert, their probability of having an acute event within the next 7 days was approximately 3-fold greater than if they had submitted an assessment without a red alert. Higher counts of red alerts on a given assessment were associated with a higher likelihood of an acute event in the next 7 days. For example, patients with 5 or more red alerts on an assessment had a 22.9% (95% CI, 16.8%-100%) probability of an acute event, nearly 8-fold greater than for patients without a red alert (Table 2).

**Table 2.** Breakout of Acute Events vs Count of Red Alerts in the Electronic Patient-Reported Outcomes Assessment in Previous 7 Days

Red alerts on assessment, No.	Scenario count, No.	Acute event in next 7 d, No. (%)	Lift above baseline (>0 red alerts rate)
0	11 955	349 (2.9)	1.0
1	1074	63 (5.9)	2.0
2	700	60 (8.6)	3.0
3	538	53 (9.9)	3.4
4	214	27 (12.6)	4.3
≥5	122	28 (22.9)	7.9

## Discussion

In this quality improvement study, we sought to determine the potential value of a daily ePRO for patients with cancer who initiated intravenous antineoplastic therapy. ePROs are increasingly viewed as a digital therapeutic because of their associations with improved cancer outcomes.^29^ Like any therapy, dose is an important consideration. Our findings illuminate some of the value a daily ePRO could provide.

### Severe Symptoms Without a Preceding Mild or Moderate Symptom

Almost half of red alerts were generated without a preceding yellow alert for the same symptom in the 7 days prior. This suggests that severe symptoms can develop without warning and a daily ePRO could facilitate addressing these symptoms in real-time to mitigate complications and relieve suffering. A weekly assessment with a 7-day recall period may not allow for this continuous, just-in-time management that could improve clinical outcomes.^11^

### Patient Symptom Fluctuations Over the Course of a Week

Over 7 days, 60.3% of fixed-time boxes showed a symptom fluctuation. Understanding trends in symptom severity could aid in improved management by informing clinicians whether interventions (eg, antiemetics, analgesics) are having the intended outcome. In our ePRO system, symptom trends were monitored using a clinician ePRO dashboard that enabled escalation and deescalation of treatments based on trends in symptoms. Further analysis is needed to better understand the clinical significance of these symptom fluctuations and how these severity changes can be optimally managed.

### Red Alert Counts and Acute Events

Symptom assessments with a red alert and red alerts per assessment were associated with an increased risk of an acute care encounter in the next 7 days. The daily cadence allowed for the capture of this data, which could be used in future prognostic analytics to triage patients in need of enhanced supportive care and to help prevent these acute events, which come at high cost to the patient and health care system.^21^ For example, we found that patients with 4 red alerts on their daily assessment had a 12.6% chance of an acute care visit within the next 7 days and were 4.3-fold more likely to have such an event than patients completing the assessment without a red alert. Unfortunately, despite the clinical team knowing of these red alert symptoms, patients sometimes ended up in the emergency department, indicating that perhaps some conditions cannot be managed remotely or that there is potentially a need for improved virtual supportive care delivery to manage these symptoms.

Not surprisingly, our study also found that patients exhibited symptoms at nearly any time, including outside clinic hours. While most assessments were completed during business hours, 42% were not, reflecting other competing demands, such as work and family, that could influence when assessments are completed. To succeed, ePROs require durable engagement by the patient, and this will likely require changes in clinical staff workflow to respond to symptom reports during and outside regular business hours to maintain patient engagement.^30^

### Limitations

There are several limitations to our study. First, our patient population reflects a single site National Cancer Institute–designated cancer center; results regarding response rate and preferences could differ with a more diverse patient population that incorporated additional sites. Second, alert threshold levels were clinically determined in consultation with nurses and oncologists. Hence, alert thresholds were subjective and potentially variable. Further refining thresholds based on their prognostic value might also improve system performance and reduce nursing effort, and this is a subject of ongoing research.^31^ Third, patients were followed longitudinally for the duration of their treatment by a centralized team whose function was to respond to their symptom reports. This centralized team could have positively biased the patients’ engagement with the ePROs and might be challenging to implement and scale in resource-constrained institutions.

## Conclusions

In this quality improvement study, we report on how daily ePROs captured symptom fluctuation in patients with cancer, including the occurrence of unanticipated severe symptoms, and their associations with at-risk moments in a patient’s cancer journey. We are in the nascent stages of using remote monitoring and digital therapeutics like ePROs to transform oncologic care. Signals from a daily ePRO can potentially guide real-time interventions to prevent adverse events, like emergency department visits and inpatient admissions. Improving ePRO delivery to optimally support patients and alert clinicians to meaningful clinical changes and coupling alerts with the right symptom management intervention in the home is the next frontier in cancer care delivery. Future studies should consider a factorial trial design to evaluate how different cadences are associated with outcomes, like quality of life, acute care visits, and overall survival, to determine the right intensity of remote symptom monitoring for different cohorts of patients during their treatment trajectory.

## Notes

Supplement. **eFigure 1.** Clinician ePRO Dashboard for Monitoring Symptom Trends **eAppendix.** InSight Care Daily Symptom Assessment **eFigure 2.** Example Patient’s Alert Levels for an Individual Symptom Within a 1-Week Period **eTable.** Symptom Severity Fluctuation Over Course of 1 Week
