# Supplementary material for: Analysis of a Remote Monitoring Program for Symptoms Among Adults With Cancer Receiving Antineoplastic Therapy
Source: JAMA Netw Open. 2022 Mar 4;5(3):e221078. doi: 10.1001/jamanetworkopen.2022.1078 (PMC8897754; doi:10.1001/jamanetworkopen.2022.1078)
Supplement: Supplement. — eFigure 1. Clinician ePRO Dashboard for Monitoring Symptom Trends eAppendix. InSight Care Daily Symptom Assessment eFigure 2. Example Patient’s Alert Levels for an Individual Symptom Within a 1-Week Period eTable. Symptom Severity Fluctuation Over Course of 1 Week [file jamanetwopen-e221078-s001.pdf]

Supplemental Online Content

Daly B, Nicholas K, Flynn J, et al. Analysis of a remote monitoring program for symptoms among adults with cancer receiving antineoplastic therapy. *JAMA Netw Open*. 2022;5(3):e221078. doi:10.1001/jamanetworkopen.2022.1078

eFigure 1. Clinician ePRO Dashboard for Monitoring Symptom Trends
eAppendix. InSight Care Daily Symptom Assessment
eFigure 2. Example Patient's Alert Levels for an Individual Symptom Within a 1-Week Period
eTable. Symptom Severity Fluctuation Over Course of 1 Week

This supplemental material has been provided by the authors to give readers additional information about their work.

© 2022 Daly B et al. *JAMA Network Open.*

eFigure 1. Clinician ePRO Dashboard for Monitoring Symptom Trends

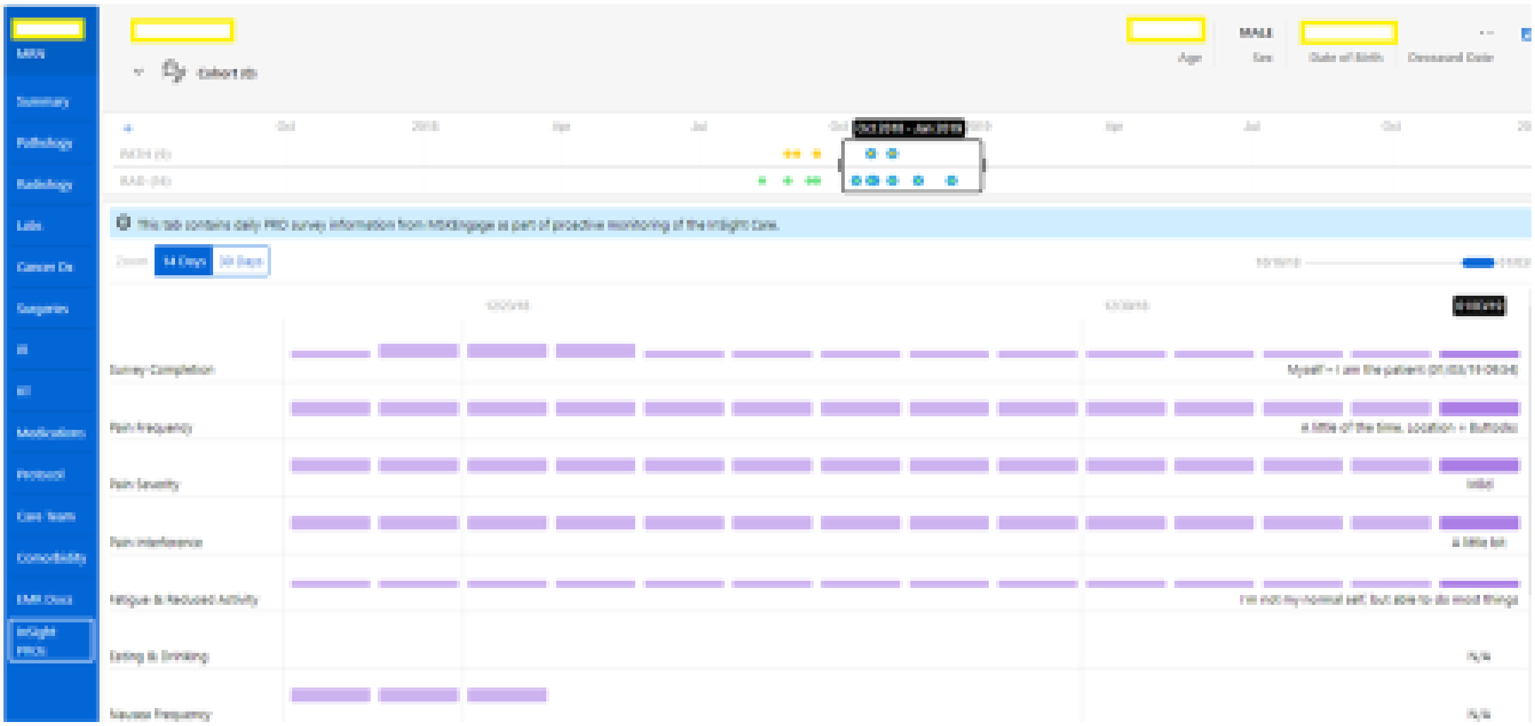

The figure depicts the technology interface the InSight Care clinical team employs to monitor an individual patient's symptoms during the enrollment period. The clinician is able to monitor how that symptom has changed in severity to determine whether an escalation or de-escalation in symptom management is indicated.

© 2022 Daly B et al. *JAMA Network Open.*

eAppendix: InSight Care Daily Symptom Assessment
All questions – no selections made:

Daily Symptom Assessment

0%

Please complete your assessment to let us know how you are feeling today.

Have you had any of the following symptoms in the past 24 hours?

Pain *	○ Yes	○ No
Fatigue or Reduced Activity *	○ Yes	○ No
Difficulty eating or drinking *	○ Yes	○ No
Nausea (feeling like throwing up) *	○ Yes	○ No
Vomiting (throwing up) *	○ Yes	○ No
Constipation (difficulty pooping) *	○ Yes	○ No
Diarrhea (loose or watery poop) *	○ Yes	○ No
Shortness of Breath *	○ Yes	○ No

© 2022 Daly B et al. *JAMA Network Open.*

In the last 24 hours, did you have any other symptoms that we have not asked you about? *

- ◯ Yes
- ◯ No

In the last 24 hours, have you gone to a non-MSK emergency room or urgent care facility, or had an unplanned hospital stay at a hospital that was not MSK? *

- ◯ Yes, a non-MSK emergency room or urgent care facility
- ◯ Yes, an unplanned hospital stay at a non-MSK hospital
- ◯ No

Who completed this survey? *

- ◯ Myself – I am the patient
- ◯ Caregiver – family member, friend, professional helper

Selections to show all other questions:

Have you had any of the following symptoms in the past 24 hours?

Pain * ◉ Yes ◯ No

How often did you have pain in the last 24 hours? *

- ◯ A little of the time
- ◯ Some of the time
- ◯ A lot of the time
- ◯ Almost all the time or all the time

© 2022 Daly B et al. *JAMA Network Open.*

Please check the boxes that correspond to areas where you have pain. *

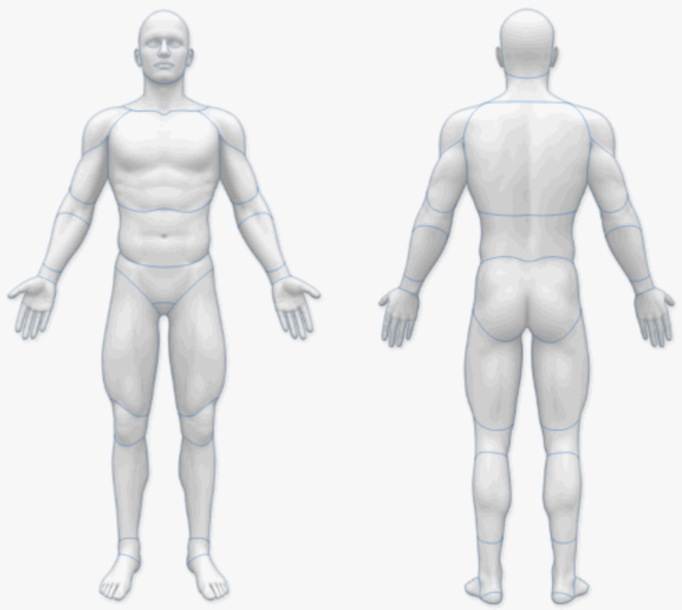

- [ ] Head
- [ ] Left Eye
- [ ] Right Eye
- [ ] Left Ear
- [ ] Right Ear
- [ ] Nose
- [ ] Mouth
- [ ] Neck
- [ ] Chest
- [ ] Abdomen
- [ ] Pelvis
- [ ] Pubis
- [ ] Left Shoulder
- [ ] Right Shoulder
- [ ] Left Arm
- [ ] Right Arm
- [ ] Left Elbow
- [ ] Right Elbow
- [ ] Left Forearm
- [ ] Right Forearm
- [ ] Left Wrist
- [ ] Right Wrist
- [ ] Left Hand
- [ ] Right Hand
- [ ] Left Thigh
- [ ] Right Thigh
- [ ] Left Knee
- [ ] Right Knee
- [ ] Left Leg
- [ ] Right Leg
- [ ] Left Ankle
- [ ] Right Ankle
- [ ] Left Foot
- [ ] Right Foot
- [ ] Back
- [ ] Loin
- [ ] Buttocks
- [ ] Left Sole
- [ ] Right Sole
- [ ] Left Back Of Knee
- [ ] Right Back Of Knee

How bad was your pain at its worst? *

- ○ Mild
- ○ Medium
- ○ Bad
- ○ Very Bad

How much did your pain make it hard to do your usual activities? *

- ○ Not at all
- ○ A little bit
- ○ Somewhat
- ○ Quite a bit
- ○ Very much

© 2022 Daly B et al. *JAMA Network Open.*

Fatigue or Reduced Activity * — (●) Yes ( ) No

How has your activity been in the last 24 hours? *

- ( ) I'm not my normal self, but able to do most things
- ( ) I'm not feeling up to most things, but in bed or chair less than half the day
- ( ) I'm able to do a little activity, but spend most of the day in bed or chair
- ( ) I pretty much stay in the bed or chair all day

Difficulty eating or drinking * — (●) Yes ( ) No

How much have you been eating or drinking in the last 24 hours? *

- ( ) The same amount as usual
- ( ) A little less than usual
- ( ) Quite a bit less than usual
- ( ) A lot less than usual or not eating or drinking at all

Nausea (feeling like throwing up) * — (●) Yes ( ) No

How often did you have nausea (feeling like you were going to throw up) in the last 24 hours? *

- ( ) A little of the time
- ( ) Some of the time
- ( ) A lot of the time
- ( ) Almost all the time or all the time

How bad was your nausea (feeling like you were going to throw up) at its worst? *

- ( ) Mild
- ( ) Medium
- ( ) Bad
- ( ) Very bad

© 2022 Daly B et al. *JAMA Network Open.*

Vomiting (throwing up) * ◉ Yes ○ No

How often did you vomit (throw up) in the last 24 hours? *

○ 1 or 2 times

○ 3 to 5 times

○ 6 or more times

Constipation (difficulty pooping) * ◉ Yes ○ No

Did you have a bowel movement (poop) in the last 24 hours? *

○ Yes

○ No

Constipation (difficulty pooping) * ○ Yes ◉ No

Diarrhea (loose or watery poop) * ◉ Yes ○ No

How many times did you have diarrhea (loose, watery poop) in the last 24 hours? *

○ 2 to 3 times

○ 4 to 6 times

○ 7 to 9 times

○ 10 or more times

© 2022 Daly B et al. *JAMA Network Open.*

Shortness of Breath *

- (selected) Yes
- No

How often have you had shortness of breath in the last 24 hours? *

- A little of the time
- Some of the time
- A lot of the time
- Almost all the time or all the time

How much did your shortness of breath make it hard to do your daily activities? *

- Not at all
- A little bit
- Somewhat
- Quite a bit
- Very much

In the last 24 hours, did you have any other symptoms that we have not asked you about? *

- (selected) Yes

  Please explain: *

- No

© 2022 Daly B et al. *JAMA Network Open.*

eFigure 2. Example Patient's Alert Levels for an Individual Symptom Within a 1-Week Period
(Individual Symptom = Pain)

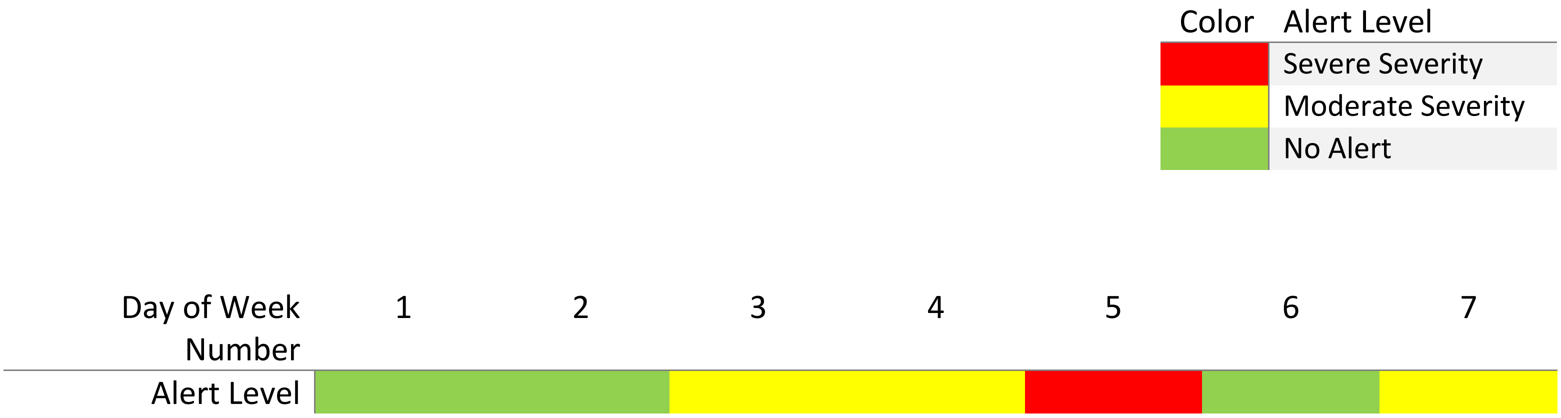

The supplemental figure demonstrates a real example of alert status fluctuation. In this real example, the patient's pain alert levels varied from no alert to yellow alert (moderate pain) to red alert (severe pain), then back down to no alert, before rising in severity again to a yellow alert (moderate pain) — during one week of monitoring.

© 2022 Daly B et al. *JAMA Network Open.*

eTable. Symptom Severity Fluctuation Over Course of 1 Week

	Scenario Description	Subgroup	Percent
1	Any Green to Yellow During Week	810	28.0%
2	Any Green to Red During Week	243	8.4%
3	Any Yellow to Green During Week	810	28.0%
4	Any Yellow to Red During Week	298	10.3%
5	Any Red to Green During Week	220	7.6%
6	Any Red to Yellow During Week	333	11.5%
7	All Symptoms Steady Green During Week	1151	39.7%
8	All Symptoms Steady Yellow During Week	0	0%
9	All Symptoms Steady Red During Week	0	0%
10	Any Fluctuation During Week	1745	60.3%

The above table demonstrates the different scenarios for how the severity of an individual symptom can fluctuate during a 7-day fixed time period. Of the **2,896** 7-day fixed time boxes in the evaluation group, 60.3% demonstrated a fluctuation in the severity of an individual symptom.

© 2022 Daly B et al. *JAMA Network Open.*
